# Synthesis of soluble melanin nanoparticles under acidic conditions using *Burkholderia cepacia* tyrosinase and their characterization†

**DOI:** 10.1039/d2ra01276f

**Published:** 2022-06-13

**Authors:** Hyun Kim, Uk-Jae Lee, Hanbit Song, Jeongchan Lee, Won-Suk Song, Heewon Noh, Min-Ho Kang, Beom-Seok Kim, Jungwon Park, Nathaniel S. Hwang, Byung-Gee Kim

**Affiliations:** School of Chemical and Biological Engineering, Seoul National University Seoul 08826 Republic of Korea byungkim@snu.ac.kr; Institute of Molecular Biology and Genetics, Seoul National University Seoul 08826 Republic of Korea; Interdisciplinary Program for Biochemical Engineering and Biotechnology, Seoul National University Seoul 08826 Republic of Korea; Bio-MAX/N-Bio, Seoul National University Seoul 08826 Republic of Korea; Institute for Sustainable Development (ISD), Seoul National University Seoul 08826 Republic of Korea; Center for Nanoparticle Research, Institute for Basic Science (IBS) Seoul 08826 Republic of Korea; Department of Biomedical-Chemical Engineering, Catholic University of Korea Bucheon 14662 Republic of Korea; Department of Biotechnology, The Catholic University of Korea Bucheon 14662 Republic of Korea

## Abstract

Melanin nanoparticles (MNPs) used for biomedical applications are often synthesized *via* the chemical auto-oxidation of catecholic monomers such as dopamine and 3,4-dihydroxyphenylalanine (DOPA) under alkaline conditions. However, the synthetic method for the chemical synthesis of MNP (cMNP) is relatively straightforward and more robust to control their homogenous particle size and morphology than the corresponding enzymatic synthetic methods. In this study, we demonstrated that the simple enzymatic synthesis of MNPs (eMNPs) with homogenous and soluble (<20 nm diameter) properties is possible using dopamine and *Burkholderia cepacia* tyrosinase (*Bc*Ty) under acidic conditions (*i.e.*, pH 3.0). *Bc*Ty was highly reactive under pH 5.0, where the natural and chemical oxidation of catechol is complex, and thus melanin was synthesized *via* the hydroxylation of phenolic substrates. The detailed chemical analysis and characterization of the physical properties of the eMNPs confirmed the higher preservation of the catechol and primary amine moieties in the monomer substrate such as dopamine under acidic conditions. The eMNPs showed enhanced antioxidant activity and conferred stickiness to the formed hydrogel compared to the chemical auto-oxidation method owing to the large number of hydroxyl groups remaining such as catechol and quinone moieties. Because of these advantages and characteristics, the synthesis of MNPs using *Bc*Ty under acidic conditions can open a new path for their biomedical applications.

## Abstract 2

Melanin nanoparticles (MNPs) used for biomedical applications are often synthesized *via* the chemical auto-oxidation of catecholic monomers such as dopamine and 3,4-dihydroxyphenylalanine (DOPA) under alkaline conditions.

## Graphical abstract

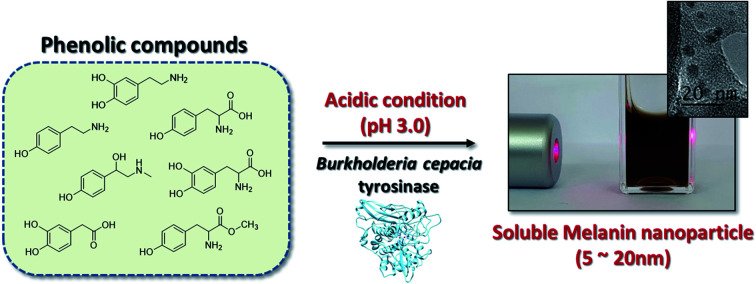

## Introduction

Melanins are natural biopolymers widely distributed in various living organisms. According to their chemical composition, melanins are classified into three types, eumelanin, pheomelanin, and allomelanin.^1–3^ The most common type of melanin present in the human body is brown-black eumelanin. Eumelanin is usually synthesized *via* the oxidation of tyrosine using the copper-containing enzyme tyrosinase, producing cyclized indole compounds such as 5,6-dihydroxyindole-2-carboxylic acid (DHICA) and 5,6-dihydroxyindole (DHI).^2,4–6^ Subsequently, eumelanin is synthesized through the spontaneous covalent and non-covalent bonding of DHICA and DHI with various precursors derived thereof.^7–9^ The eumelanin in living microorganisms and animals can react and form covalent bonds with amino acids such as lysine, histidine, and cysteine or proteins during its synthesis, resulting in the formation of nano to micro-size insoluble particles (25 nm to a few μm) with broad size distributions in cells.^10,11^ However, the broad size distribution of melanin particles limits their applications, given that they do not have a well-distributed colloidal dispersion.

Thus, MNPs synthesized from a single substrate *via* the chemical method with a similar size and morphology (cMNP) are more desirable for industrial and biomedical applications.^12–15^ Polydopamine nanoparticles (herein called cMNP) synthesized using dopamine monomer *via* auto-oxidation under alkaline conditions have a relatively narrow particle size distribution.^16,17^ However, cMNPs are somewhat insoluble, and the synthesis of cMNPs with an average diameter of less than 50 nm is difficult. Previous studies have shown that the regulation of the subsequent formation of agglomerates and melanin polymerization rate following the dopamine oxidation reaction affects the formation of homogenous melanin nanoparticles. Thus, to effectively narrow the size distribution of cMNPs during the polymerization processes the following strategies have been employed: (i) varying the concentration of radical initiator/scavenger such as plasma-activated water,^18^ transition metal catalyst,^19^ potassium manganite,^4^ 2-phenyl-4,4,5,5-tetramethyl imidazoline and edaravone,^20^ (ii) UV irradiation,^21^ and (iii) high temperature and hydrothermal pressure under acidic conditions.^22^ However, the enzymatic method cannot control the synthesis of MNPs like the simple chemical method. Therefore, we speculated that if the cross-linking of a phenolic substrate inhibits the enzymatic melanin synthesis reaction, it can help produce different forms of eMNPs rather than large aggregates of cMNPs and eMNPs.

We noticed that in nature, mussels regulate the degree of crosslinking among DOPA moieties on the mussel foot protein (Mfp) by ambient pH changes to maintain their stickiness for surface adhesion. Mussels in seawater secrete Mfp with a high DOPA content (∼30 mol%) to adhere to the surface of ship bottoms or rocks to survive. DOPA is known to be generated by post-translational modification of Mfp *via ortho*-hydroxylation at its tyrosine residues.^23,24^ Mussels use hydrogen bonding and metal chelation between the catechol residues of DOPA and the surface of target materials.^23,25^ To maintain a high DOPA content, mussels have to change the pH of Mfp to a more acidic condition (pH 2.0–5.0) against ambient seawater (pH 8.0). As a result of lowering the pH around Mfp, auto-oxidation of the catechol residue of DOPA is suppressed, and its crosslinking with the amine or thiol groups of highly reactive amino acids is prevented. Based on this observation, we hypothesized that maintaining a high DOPA content in Mfp is possible by only controlling the acidic pH conditions of the Mfp expression. Then, eumelanin-like nanoparticles synthesized under these acidic conditions are expected to be somewhat different from the large aggregates of melanin synthesized by enzymes under neutral to alkaline conditions generally found in *in vitro* reaction systems. Therefore, tyrosinase reactions under acidic conditions were examined to support our hypothesis.

Previously, we identified *Burkholderia thailandensis* tyrosinase (*Bt*Ty, AWY65947.1) having optimal activity at pH 5.0 (ref. 26) and tried to find new tyrosinases having optimal activity at pH lower than 5.0. Recently, based on the amino acid sequence of *Bt*Ty, we identified *Burkholderia cepacia* tyrosinase (*Bc*Ty, SEU13261.1) having activity even at pH 3.0. Herein, to prove our hypothesis and better understand the biosynthesis of melanin, we synthesized homogenous but also soluble eMNPs from dopamine using the newly found tyrosinase, *Bc*Ty. For comparison, both cMNPs obtained from dopamine *via* auto-oxidation under acidic to alkaline conditions and eMNPs synthesized from dopamine using tyrosinase in alkaline solution were used as controls. The physicochemical properties of eMNPs and cMNPs were compared using various tools such as UV-vis spectroscopy, mass spectroscopy, XPS, SEM, TEM, and FTIR. Based on this study, we could better understand how homogenous and water-soluble eMNPs can be synthesized by the enzymatic method under acidic conditions, where the auto-oxidation of catechol moiety is suppressed. Then, we applied the synthesis of soluble eMNPs to various other applications using the optimized tyrosinase reaction and various substrates such as tyramine, tyrosine methyl ester (TME), 3,4-dihydroxyphenyl acetic acid (DOPAC), and synephrine.

## Experimental

### Materials

NcoI and HindIII restriction enzymes were purchased from Thermo Scientific. Luria-Bertani (LB) broth and LB agar were purchased from BD Difco (Sparks, USA). Ni-NTA agarose was purchased from Qiagen (Hilden, Germany). Acetic acid and Amicon 0.5 mL 3k filter were purchased from Merck (Darmstadt, Germany). Dopamine hydrochloride, copper sulfate, and all other chemicals were purchased from Sigma-Aldrich Korea (Seoul, Republic of Korea). *Burkholderia cepacia* (ATCC 25416) was purchased from the Korean Agricultural Culture Collection (KACC, Wanju, Republic of Korea).

### Plasmid construction

For the construction of plasmid-expressing tyrosinase, pET28a was used as in the previous report^26^ and produced through the circular polymerase extension cloning (CPEC)^27^ method. The list of primers is presented in Table S1.† Genomic DNA for cloning was extracted from *Burkholderia cepacia* (*Bc*) using a G-spin genomic DNA extract kit (Intron, Republic of Korea), and PCR was performed to amplify the *Bc*Ty gene (gi: 1095297873) with the C-terminal His-tag inserted into the pET28a vector using NcoI and HindIII restriction enzyme sites.

### Expression and purification of *Bc*Ty

For the expression of *Bc* tyrosinase (*Bc*Ty), the expression vector was transformed into *E. coli* BL21 (DE3) by heat shock treatment, and the transformed strain was selected on an LB agar plate with the antibiotic selection marker kanamycin (50 μg mL^−1^). A single colony was inoculated in a test tube with 3 mL of the same LB broth with kanamycin, and the cells were cultured in a shaking incubator at 37 °C and 200 rpm overnight. Subsequently, 500 μL of cultured cells was transferred to 50 mL of fresh LB medium with antibiotic markers, and then the cells were grown to approximately 0.6 at OD600. Furthermore, the protein expression was induced by adding 0.1 mM of IPTG and 1.0 mM of CuSO_4_. After 20 h at 37 °C culture, the cells were harvested by centrifugation at 4000 rpm for 20 min. The cell pellets were resuspended in 50 mM pH 8.0 Tris buffer and disrupted by ultrasonication. After ultracentrifugation at 16 000 rpm for 30 min, the supernatant was collected. The supernatant was applied to the Ni-NTA agarose column for enzyme purification. Subsequently, the cell lysate was applied to the Ni-NTA column after pre-equilibrium with 50 mM pH 8.0 Tris buffer with 5.0 mM imidazole and 300 mM NaCl buffer. The bound proteins were washed with 50 mM pH 8.0 Tris buffer with 30 mM imidazole and 300 mM NaCl buffer. Tyrosinase was eluted with 50 mM pH 8.0 Tris buffer and 250 mM imidazole. The concentration of purified enzyme was measured using the Bradford assay.

### Synthesis of eumelanin-like nanoparticles

The synthesis of the chemically synthesized eumelanin-like nanoparticles (cMNP) was performed using the previously reported method.^16,17^ Briefly, 3 mg mL^−1^ of MNP substrate including tyrosine, dopamine, 3,4-dihydroxyphenylalanine (DOPA), 3,4-dihydroxyphenylacetic acid (DOPAC), synephrine, and tyrosine methylester were dissolved in 2 mL of 50 mM pH 8.5 Tris buffer in a clear glass bottle. The oxidation reaction was initiated at 37 °C with 200 rpm agitation for 12 h. For further analysis, the MNP aggregates were collected by centrifugation at 4000 rpm for 10 min and washed three times with distilled water (DW).

For the synthesis of the enzymatically synthesized eumelanin-like nanoparticles [eMNP-*i*, (*i* = 3–5)], various pH solutions of acetic acid buffer were prepared. Firstly, 100 mM acetic acid was added to DW and NaOH was added to adjust to the desired pH (pH 3.0, 4.0, and 5.0). Next, 200 nM of *Bc*Ty and 10.0 μM of CuSO_4_ tyrosinase Cu^2+^ cofactor were added to 3 mL of acetic acid buffer in a glass bottle. Then, 3 mg mL^−1^ of MNP substrate dissolved in DW was added to the reaction solution. eMNPs were synthesized at 37 °C with 200 rpm agitation for 12 h. eMNP-3PEG was synthesized under the same reaction conditions as eMNP-3s. Briefly, 3 mg mL^−1^ of dopamine was added to the reaction mixture and left to react overnight to prepare eMNP-3. Next, 1.0 mg mL^−1^ of terminal thiol-modified polyethylene glycol (PEG-SH) was added to 100 mM pH 3.0 acetic acid buffer, and then eMNP-3PEG was synthesized at 37 °C with 200 rpm agitation for 12 h. eMNP-3PEG was filtered with an Amicon Ultra 0.5 mL 3k filter to remove the unreacted PEG-SH.

### Instrumental analysis

The UV-vis spectra of cMNP and eMNPs were measured using a SPECTROstar nano UV-vis spectrophotometer (BMG LABTECH, Germany) in the wavelength range of 220 nm to 1000 nm. The average hydrodynamic diameter and zeta potential of the MNPs were measured *via* dynamic light scattering (DLS) using a Zetasizer Nano-ZS system (Malvern Instruments, Inc., UK). The particle size of cMNP and eMNPs was measured *via* field-emission scanning electron microscopy (FE-SEM). cMNP and eMNPs were freeze-dried and platinum-coated by sputtering before the FE-SEM analysis. The morphology and size of the samples were analyzed on an FE-SEM 7800 Prime (JEOL Ltd., Japan) at the National Center for Inter-University Research Facilities (NCIRF) at Seoul National University with an accelerating voltage of 5.0 kV. The particle size was analyzed using the ImageJ software. The particle size measurement and physical characterization of the soluble eMNP-3 were performed *via* transmission electron microscopy (TEM, JEM-2100F, JEOL Ltd., Japan) and Cs-corrected transmission electron microscopy (Cs-TEM, JEM-ARM200F, JEOL, Japan) with an accelerating voltage of 200 kV. The soluble eMNP-3 was placed on a TEM grid (lacey carbon grid, 3 mm, 300 mesh) and dried before the TEM analysis. The particle size and gap between the onion-like stacking structures were analyzed using the ImageJ software. Attenuated total reflectance Fourier transform infrared (FTIR/ATR) analysis was performed using a TENSOR27 (Bruker, Germany) at the NCIRF at Seoul National University. All MNP samples were freeze-dried before the FTIR/ATR analysis. Scans were acquired in the wavenumber range of 4000 cm^−1^ to 400 cm^−1^. The peak resolution was 0.4 cm^−1^ and the number of scans was 32. Baseline correction for each sample was performed using OriginPro 2020b. X-ray photoelectron spectroscopy (XPS) analysis was performed on a UHV Multipurpose Surface Analysis System (Sigma Probe, Thermo, UK) operating at base pressure of under 10^−9^ mbar. Each photoelectron spectrum was excited using an Al Kα (1486.6 eV) anode operating at a constant power of 105 W (15 kV and 10.0 mA). The cMNP and eMNP samples (*i.e.*, 100 μL) were dried on a 1 cm × 1 cm silica wafer under vacuum conditions before the XPS analysis.

### Evaluation of the antioxidant activity of the soluble MNP

The antioxidant activity of MNPs was studied using a 2,2-diphenyl-1-picrylhydrazyl (DPPH) color change assay known as the free radical scavenging activity assay.^28,29^ For the DPPH assay, ascorbic acid, cMNP, eMNP-3, and eMNP-3PEG were compared. For this experiment, 0.2 mM of fresh DPPH solution in methanol was prepared. Firstly, various amounts (0 to 14 μL, final concentration of 0 to 0.07 mg mL^−1^ in 200 μL) of antioxidants were divided in 100 μL of pH 7.4 PBS buffer in a 96-well plate. Then, 100 μL of DPPH solution was added to make the reaction mixture with the final concentration of 0.1 mM of DPPH. The reaction mixture was incubated for 20 min at 37 °C under dark conditions. After the incubation was completed, the absorbance of the solution against the blank was measured at 517 nm. The following equation was employed to calculate the radical scavenging activity.
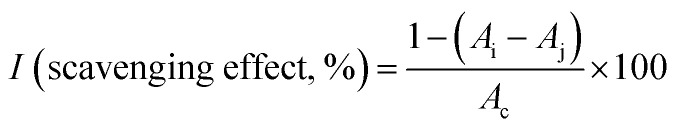
where *A*_i_ is the absorbance of the test sample, antioxidant and DPPH solution, *A*_j_ is the absorbance of the antioxidant solution without DPPH as control, and *A*_c_ is the absorbance of the DPPH solution without antioxidant. EC_50_ refers to the concentration at which the antioxidant can scavenge radicals, corresponding to 50% of the DPPH concentration, which was calculated using the non-linear regression method and GraphPad Prism 7.0.

### Synthesis of sticky eMNP–gelatin hydrogel and characterization

For the preparation of the sticky hydrogel, 10.0 wt% of gelatin was dissolved in 100 mM acetic buffer (pH 3.0) at 40 °C. After gelatin was entirely dissolved and the bubbles in the reaction solution were removed by sonication, 3 mg mL^−1^ of dopamine and 10.0 μM of CuSO_4_ were added to 2 mL of gelatin (10.0 wt%) solution. Dopamine was not included in the control hydrogel sample. The gelatin mixture was incubated in a water bath at 37 °C for 15 min. Next, 1.0 μM of *Bc*Ty was added to the mixture to initiate the crosslinking reaction between gelatin and eMNP. Then, 190 μL of the reaction mixture was crosslinked in a customized polydimethylsiloxane (PDMS) mold with a diameter of 16 mm and thickness of 5 mm overnight at 37 °C.

The mechanical properties of the hydrogel were measured using a Universal Testing Machine (UTM, 100 N of the load cell, EZ-SX STD, Shimadzu, Japan).^52^ After an overnight crosslinking reaction, the hydrogel samples were placed on the sample table. The probe was compressed vertically at a distance of 2 mm from the top of the hydrogel to measure the adhesion force. After 10 s, the probe was lifted to the upward position at a speed of 3 mm min^−1^ to measure the adhesion force of the hydrogel attached to the table surface. Each hydrogel was compressed vertically at a speed of 3 mm min^−1^ to measure its mechanical strength and the Young's modulus was calculated from UTM strain–stress curve data.

## Result and discussion

### Production of soluble eMNPs at acidic pH using *Bc*Ty

**Fig. 1:**
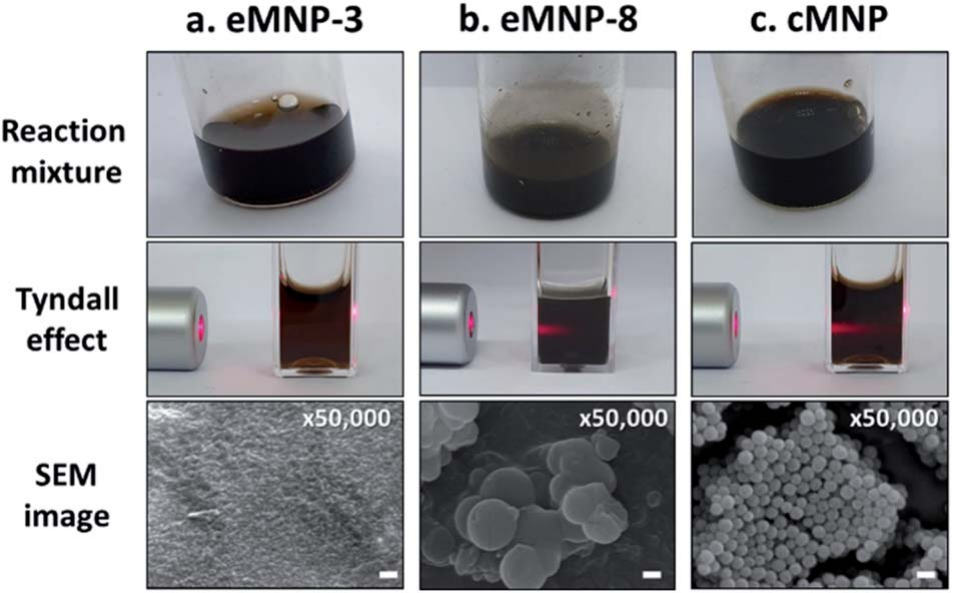
Digital pictures of melanin reaction solutions synthesized from 3 mg mL^−1^ of dopamine using various synthesis methods, Tyndall effect by irradiating 660 nm red laser on the solutions and their scanning electron microscopy images: (a) pH 3.0 – *Bc*Ty 200 nM, (b) pH 8.0 – *Bm*Ty 200 nM, and (c) pH 8.5 – chemical auto-oxidation method using Tris base. Scale bar: 200 nm. The samples for Tyndall effect observation were diluted with water from 3 mg mL^−1^ to 0.05 mg mL^−1^.

Based on our hypothesis that soluble eMNPs with a high catechol-moiety content can be synthesized under acidic conditions, we identified *Bt*Ty and *Bc*Ty, tyrosinases with activity even at pH 5.0 or lower, by heterogeneous expression system of *E. coli*. *Bc*Ty purified through His-tag had the highest enzyme efficiency (*k*_cat_/*K*_M_) for l-tyrosine and l-DOPA among the bacterial tyrosinases reported thus far (Table S2†), which showed optimal activity at pH 4.0 and activity even at pH 3.0 (Fig. S1†). Thus, eMNP was produced from 3 mg mL^−1^ of substrate by 200 nM of *Bc*Ty (0.012 mg mL^−1^) with 10 μM of CuSO_4_ as the tyrosinase cofactor in 3 h. We synthesized eMNPs from dopamine using *Bc*Ty at pH 3.0 to 5.0 [named eMNP-*i* (*i* = pH value)] and compared their physicochemical properties with that of the controls, *i.e.*, eMNP-8 and cMNP synthesized under alkaline conditions (Fig. 1).

To investigate the characteristics of eMNPs synthesized from dopamine under acidic conditions of pH 3.0 to 5.0, eMNP-8 was synthesized using *Bacillus megaterium* (*Bm*) tyrosinase, which exhibited higher activity than *Agaricus bisporus* tyrosinase at neutral to alkaline pH,^30^ to compare the properties of eMNPs according to the reaction pH. In addition, as a control, the auto-oxidation of dopamine (p*K*_a_ = 8.93 (ref. 31)) cannot occur under acidic conditions (pH 3.0 to 5.0), and thus cMNP was obtained by oxidizing 1 mg mL^−1^ dopamine (6.53 mM) using ammonium persulfate (APS), a strong chemical oxidizing agent. However, cMNPs were not synthesized even when the same mass concentration of APS was used (*i.e.*, 1 mg mL^−1^, 4.38 mM) as dopamine, under acidic conditions at pH 3.0 to 5.0 (Fig. S2†). The reaction mixture was subjected to the oxidation reaction for a longer time, such as 24 h, which displayed a red-brown color due to the formation of melanin-like insoluble particles (Fig. S2a–c†). However, the UV spectra of cMNP-3, 4, and 5 indicated that most of the dopamine was not completely oxidized to quinone and remained in the reaction mixture (Fig. S2d†). To compare the properties of cMNPs synthesized through complete oxidation of the substrate, we compared the physicochemical properties of eMNPs synthesized from *Bc*Ty with that of cMNP synthesized under basic conditions, similar to many previous studies.

For both cMNP and eMNP-8, insoluble MNPs could be observed with the naked eyes, whereas eMNP-3 synthesized at pH 3.0 was completely soluble (Fig. 1). When a red laser light (640–660 nm) was irradiated on the reaction mixture, the Tyndall scattering effect was not observed only in eMNP-3, suggesting that soluble MNPs were synthesized under this condition.^32^ The SEM image of the homogenous eMNP-3s displayed an average particle size of *ca.* 7 nm diameter with a narrower size distribution than that of cMNPs (Fig. 1d and e and S3a†). Moreover, the TEM images of the eMNP-3 reaction solution showed that it contained onion-like pi–pi stacking structures among the phenolic moieties usually found in eumelanin-like nanoparticles (Fig. S3b†).^33^ In contrast, the eMNP-4, -5, and -8 samples tended to produce rather heterogeneous and large micron-sized agglomerates, generating insoluble MNP reaction mixtures (Fig. 1b and S4†).

In general, when dopamine is spontaneously oxidized at neutral to alkaline pH, it immediately forms an indole-ring structure *via* rapid ring cyclization and shows strong absorbance at 475 nm, indicating the formation of dopachrome.^4,34,35^ Given that the auto-oxidation of dopamine is suppressed in an acidic environment, *i.e.*, pH 3.0 to 5.0, the solution was colorless (data not shown). However, when *Bc*Ty was added to the pH 3.0 reaction mixture, the dopamine oxidation reaction was initiated, and the solution immediately turned yellow, then red, and gradually changed to brown-black color upon the termination of the reaction after 3 h (Fig. S5a†). The UV spectrum change profile shows that dopamine was oxidized immediately after the reaction started, and the yellow dopamine quinone (*λ* = 390 nm) was formed within 15 min (Fig. S5b†). Given that the decay rate of dopamine quinone is strongly pH-dependent,^36^ the production rate of aminochrome by the subsequent cyclization of dopamine quinone diminished. Accordingly, aminochrome (*λ* = 475 nm), a cyclization product of dopamine quinone, exhibited the maximum absorbance after 60 min of initiation reaction under acidic conditions.^9,21,34,37–39^ As the reaction proceeded, the oligomeric structure of MNP was formed, gradually showing a broadband absorption in the light spectrum, and the color of the product became black. After completion of the reaction, the reaction mixture appeared dark brown and soluble eMNP-3 was generated in the presence of *Bc*Ty.

To confirm that the synthesis of the soluble eMNP at acidic pH (*i.e.*, *ca.* pH 3) is a general phenomenon for the synthesis of soluble eMNPs using other phenolic substrates such as monophenolic-, diphenolic-, and catecholamine-type compounds, the further synthesis of eMNP using *Bc*Ty was performed at pH 3. All the phenolic substrates, including tyramine, tyrosine, DOPA, tyrosine methyl ester (TME), 3,4-dihydroxyphenylacetic acid (DOPAC), and synephrine, produced soluble and evenly dispersed eMNPs under this condition (Fig. S6 and S7†). In addition, the digital image and UV spectra of the reaction solutions of eMNP synthesized from various phenolic substrates, including dopamine, showed that the substrates could be oxidized to form eumelanin-like particles, suggesting that the synthesis of the soluble eMNP-3s is a general phenomenon for various substrates at acidic pH.

### Understanding the synthetic mechanism of soluble eMNP-3 through chemical structure and functional group analysis

**Fig. 2:**
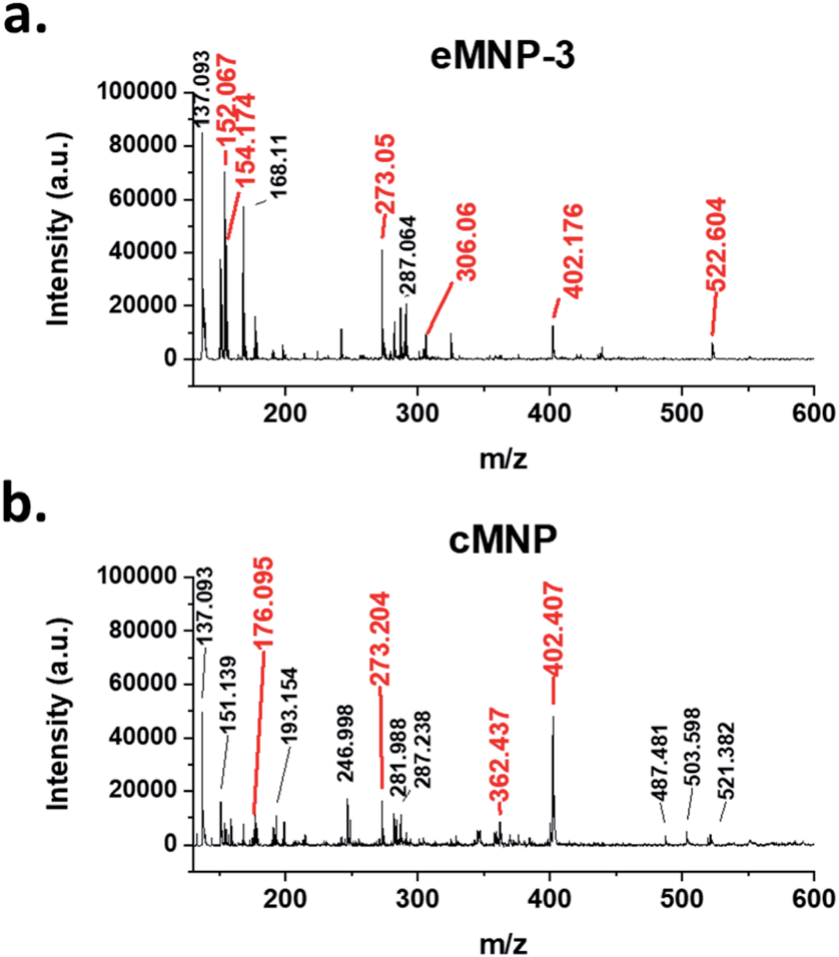
Comparison of MALDI-TOF mass spectra of (a) eMNP-3 and (b) cMNP. The mass values indicated by the red line and red text are values that can predict the chemical structure, and the expected chemical structure is indicated in ESI, Fig. S8.†

To better understand how eMNP-3 has a homogeneous particle size distribution like cMNP but is soluble in water, a detailed analysis was carried out on its chemical structure and surface properties. Although the well-defined chemical structure of MNPs from dopamine cannot be easily identified, the structural details of their oligomeric precursors have been reported using mass spectrometry.^5,40,41^ Thus, the MALDI-TOF spectra of eMNP-3 and cMNP were compared for the structural analysis (Fig. 2). Here, the MNP precursors up to the maximum trimers synthesized from dopamine ([M + H]^+^ = 273.20, 305.34, 402.39, 522.75, Fig. 2a and b and S8†) and unknown dopamine tri- and tetramer precursors (*m*/*z* = 487.48, 503.60, and 521.38, Fig. 2b) were annotated.^5,40,41^ There were no significant differences in the oligomeric chemical structure of the MNPs. However, in the mass spectra of eMNPs, unlike that of the cMNPs, the dopamine monomer ([M + H]^+^ = 154.09, Fig. 2a and S8†/[M + Na]^+^ = 176.07, Fig. 2b and S8†) and dopamine quinone ([M + H]^+^ = 152.07, Fig. 2a and S8†) were detected, suggesting the presence of non-cyclized and/or non-cyclized monomers in eMNPs.

**Fig. 3:**
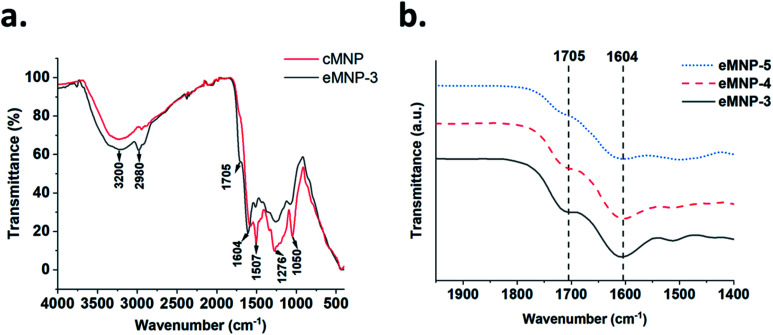
(a) Comparison of the FTIR spectra of eMNP-3 and cMNP. (b) Increasing trends of C=O stretching and N–H bending were observed according to the decrease in the synthesis of eMNP at the pH of 5.0 to 3.0.

Further, ATR-FTIR spectroscopy was used to identify the functional groups on cMNPs and eMNPs using dry powder of cMNPs and eMNPs (Fig. 3). The broad peaks in the range of 3600–2800 cm^−1^ in the FTIR spectra of cMNP and eMNP-3 (Fig. 3a) correspond to the O–H stretching in both MNPs.^20^ The peaks that can be annotated as indolestructures appeared in the range of 1600–1050 cm^−1^ (ref. 40) (Fig. 3a). In the FTIR spectra of both eMNP-3 and cMNP spectra, the absorption of the benzene ring C–C or –NH in the heterocycle ring was observed at 1507 cm^−1^ and that for the hydroxyindole O–H group at 1276 cm^−1^.^42,43^ In addition to the peak for hydroxyindole, in which the dopamine substrate was ring-cyclized, eMNP-3 showed somewhat smaller absorption peaks at 1507 cm^−1^ and 1276 cm^−1^ than that of cMNP, indicating the presence of a non-cyclized hydroxyindole monomer. The absorptions of the aliphatic carbon peak at 2980 cm^−1^, the aromatic C=C or C–O of the benzene ring of dopamine at 1604 cm^−1^ and the carbonyl C=O bond peak of hydroxyquinone at 1705 cm^−1^ were observed in the spectrum of eMNP-3.^44,45^ The absorption intensity of the peaks at 1705 and 1604 cm^−1^ increased with a decrease in the reaction pH during the synthesis of eMNP (Fig. 3b). An intermolecular covalent bond was formed by the Michael addition and Schiff base reaction between the quinone-type melanin precursors generated after the dopamine oxidation reaction, and if intramolecular cyclization increased, the absorption of the indole ring-related peak would increase. Therefore, the presence of higher absorbance peaks corresponding to the aliphatic C–H and C=O stretching in the FTIR spectrum of eMNP-3 than that in the spectrum of cMNP suggests the presence of a non-cyclized dopamine monomer.^44^

**Fig. 4:**
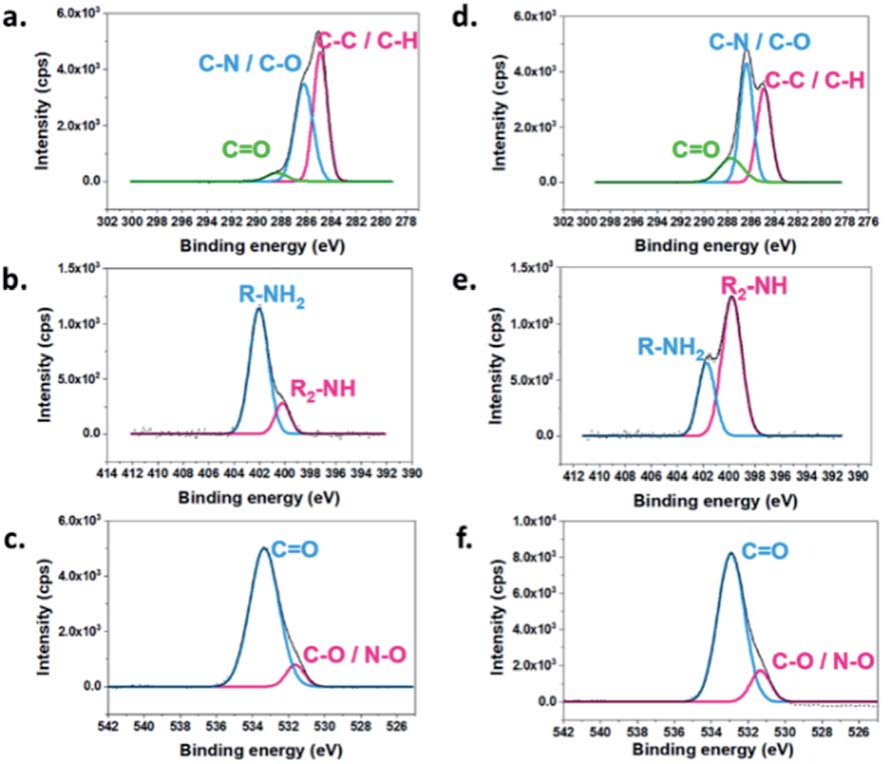
XPS spectra showing the C 1s, N 1s and O 1s peaks of eMNP-3 and cMNP. (a) C 1s of eMNP-3, (b) N 1s of eMNP-3, (c) O 1s of eMNP-3, (d) C 1s of cMNP, (e) N 1s of cMNP, and (f) O 1s of cMNP.

To specify the functional groups of the non-cyclized monomer, the surface element compositions and chemical state of eMNP-3 and cMNP were analyzed and compared through XPS (Table 1, Fig. 4 and S9†).^22,46^ In the spectrum of eMNP-3, the peaks for the C–N and C–O bond decreased, whereas the peaks attributed to the C–C (sp^1^) and C–H bonds increased (Table 1a and Fig. 4a and d). In addition, according to the O 1s elemental composition data of eMNP, the peak area ratio of the C=O functional groups to C–O/N–O bonds increased slightly to 6.81 (= 87.2/12.8) (Table 1c and Fig. 4c) compared to that of cMNP, *i.e.*, 4.03 (= 80.1/19.9) (Table 1c and Fig. 4f). The C 1s and O 1s deconvoluted spectra showed that cMNPs had the most covalently cross-linked structure among the MNP precursors with a cyclized indole structure compared to eMNPs.^22,46^ The deconvoluted C 1s, N 1s and O 1s spectra, together with the mass analysis and FTIR spectra consistently suggest that more acyclic monomers remained in eMNP-3. The primary amines remaining without cyclization in the chemical structure of the dopamine monomer could be confirmed by the deconvoluted N 1s spectrum.^22,39^ The primary amine peak in the XPS spectra (R–NH_2_, 402.03 eV) substantially increased in eMNP-3 to 81.0% compared to 32.0% in cMNP (Table 1b and Fig. 4b and e). In addition to the inhibition of self-cyclization of dopamine under acidic reaction conditions below pH 5, deprotonation of the primary amine group of dopamine was also inhibited. Consequently, the particle size of less than 20 nm for eMNP-3 is attributed to the critical effect of the low pH condition. Although dopamine quinone was rapidly formed by the added tyrosinase, given that acidic conditions inhibited additional cyclization, the content of residual dihydroxy and quinone forms of dopamine derivatives participating in the MNP polymerization was relatively high. Due to the abundance of non-cyclized dopamine derivatives and the presence of a large amount of positively charged primary amines under neutral pH storage solution (*e.g.*, pH 7.4 PBS), eMNP-3 could be expected to have a higher surface charge potential than cMNP.

**Table 1:** XPS peak binding energy assignments to (a) C 1s, (b) N 1s and (c) O 1s functional groups and peak area ratio for each functional group

Chemical bond (binding energy, eV)	eMNP-3	cMNP
**(a) C 1s (area, %)**		
C–C and C–H (284.87)	50.0	35.0
C–N and C–O (286.21)	48.4	57.9
C=O (288.37)	1.6	7.1

**(c) O 1s (area, %)**		
C–O/N–O (531.62)	12.8	19.9
C=O (533.35)	87.2	80.1

**(b) N 1s (area, %)**		
R_2_–NH (400.17)	19.0	68.0
R–NH_2_ (402.03)	81.0	32.0

### Characterization of surface zeta potential properties of eMNPs

**Fig. 5:**
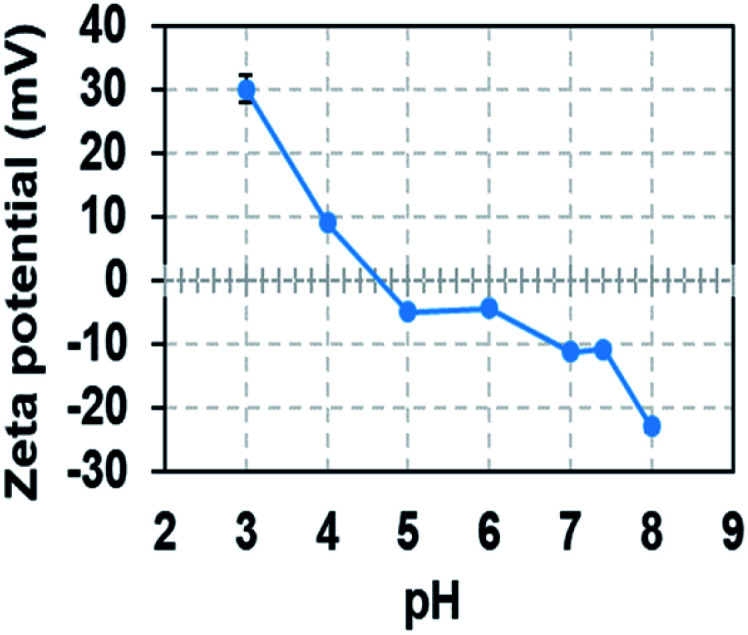
Changes in zeta potential of eMNP-3 with a variation in pH of storage buffer. All the samples were diluted with storage buffer from 3 mg mL^−1^ to 0.05 mg mL^−1^.

Melanin nanoparticles generally appear to have negatively charged surface properties due to the free radicals generated by the *o*-quinone moieties on their surface. The zeta potential of eMNP-3s at pH 3.0 was *ca.* +30.13 mV, but this value changed, and was eventually determined by the pH of their storage buffer solution. Therefore, the zeta potential of eMNP-3s in DW displayed a negative value (*i.e.*, −6.51 mV) but smaller than that of cMNPs (−34.4 mV). eMNP-3s dissolved in pH 7.4 PBS buffer had a potential of −10 mV (Table 2), suggesting that the surface zeta potential values of eMNP-3s are somewhat negatively proportional to the increase in pH of their storage solution (Fig. 5).

**Table 2:** Average hydrodynamic diameters and zeta potential of eMNP-3, 4, 5 and cMNP measured by DLS

	Hydrodynamic diameter (nm)	Zeta potential^a^ (mV)
eMNP-3	7.13 ± 0.97	−6.51
eMNP-4	400.8 ± 32.59	−9.15
eMNP-5	618 ± 42.07	−12.8
cMNP	153.96 ± 19.71	−34.4

a All the samples for zeta potential measurement were diluted to 0.05 mg mL^−1^ using PBS (pH 7.4).

It is well known that when the surface charge of colloidal nanoparticles is within the range of −5 to +5 mV, the electrostatic attraction among the particles tends to induce particle agglomeration. Given that the surface zeta potential of eMNP-3s at physiological pH (*i.e.*, pH 7.4) gradually tended to decrease to *ca.* −10 mV, electrostatic repulsive forces become dominant, and thus they are readily dispersible and stable enough to be applied under biological conditions without any problems. However, eMNP-3s in pH 7.4 PBS buffer slowly aggregated within four weeks of storage and completely precipitated (Fig. S10a†). Thus, to improve the dispersity of the soluble eMNP-3s under physiological conditions, surface coating with terminal thiol-modified polyethylene glycol (PEG-SH) was carried out following a previous report (Fig. S10b†).^17,47^ The resulting surface PEGylated eMNP-3s had a larger hydrodynamic diameter of 32.67 ± 12.72 nm (Fig. S10c†), and the soluble eMNP-3PEGs were well dispersed and stably maintained in pH 7.4 PBS buffer for more than three months under ambient conditions (data not shown).

### Evaluation of antioxidant effect of soluble and ultra-small eMNP

The exact cause of the action of melanin as a radical scavenger is difficult to conclude, although various causes are known. However, as one of the important factors, melanin is considered a very good antioxidant biomaterial because its subunits contain a significant amount of a catechol and quinone moieties, which can act as both an electron acceptor and a donor.^48^ Therefore, as the contact surface with the radical scavenging functional groups located on the MNP surface increases, the surface area of the nanoparticles decreases, and their antioxidant effect also increases.^16,20,49^ Given that eMNP-3s are nanoparticles with a diameter of less than 10 nm, they can show high free radical scavenging activity owing to their high surface-to-volume ratio (Table 3). In addition, similar to the previous functional group analysis, it can be expected that the radical scavenging activity further increases by the catechol and quinone forms of dopamine, which play an essential role in the scavenging of radicals present due to their significant content in eMNPs. According to the analysis of the reduction of DPPH concentration in the reaction mixture, the antioxidant activities (EC_50_) of cMNPs and eMNP-3s were estimated to be 30.86 μM and 8.95 μM, respectively (Table 3 and Fig. S11†). The EC_50_ of eMNP-3PEGs, which were PEGylated to further improve the dispersity of eMNP-3s, was 2.99 μM, showing antioxidant activity similar to that of ascorbic acid (2.98 μM).

**Table 3:** Comparison of the radical scavenging activities (EC_50_) of ascorbic acid and three MNPs on DPPH

	EC_50_ (μmol L^−1^)
Ascorbic acid	2.98
eMNP-3	8.95
eMNP-3PEG	2.99
cMNP	30.86

### Mussel foot protein (Mfp)-inspired sticky hydrogel in acidic condition

Based on the characterization of eMNP-3, it was revealed that the MNPs synthesized with dopamine at low pH have a significant content of catechol and protonated primary amine groups like Mfp, which mediates the attachment of mussels to solid surfaces such as rocks and the bottom surface of ships.^23–25^ Consequently, if a hydrogel is made of these eMNP-3s, its adhesive forces can be enhanced by the high contents of dihydroxy functional groups and primary amines to gelation materials.

**Fig. 6:**
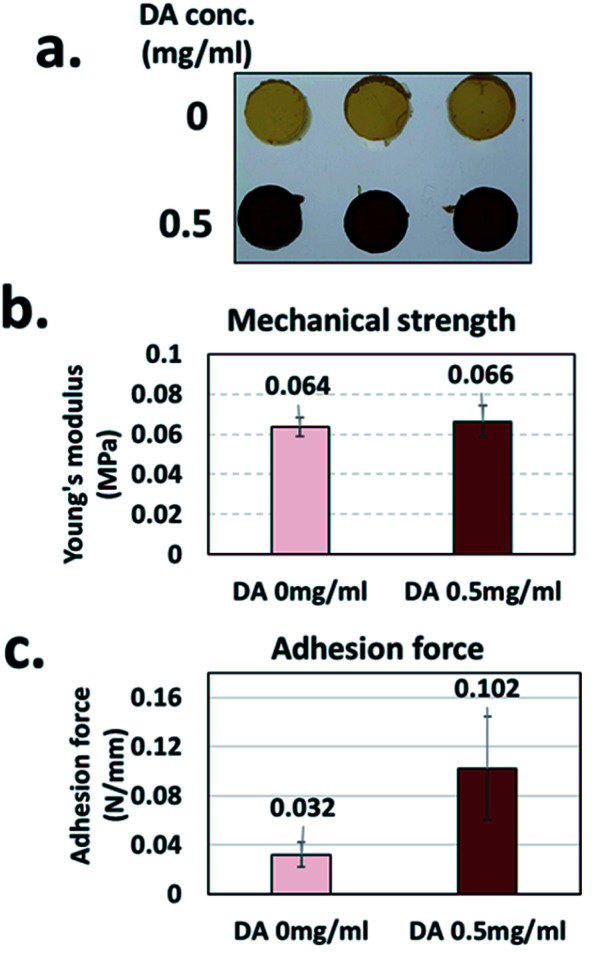
(a) Representative image of gelatin–eMNP hydrogel according to the added dopamine (DA) concentration. (b) Mechanical properties of the control gelatin hydrogel and gelatin–eMNP-3 hydrogel. (c) Adhesion force of the gelatin hydrogel and gelatin–eMNP-3 hydrogel.

When a biocompatible gelatin is used as a gelation base material, the tyrosine residues present in gelatin (*i.e.*, less than 0.5%)^50^ can partially participate in the oxidation and subsequent crosslinking reactions by *Bc*Ty. We prepared two gelatin hydrogels with and without eMNP-3s synthesized from dopamine using *Bc*Ty, yielding two hydrogels with a size of 0.8 cm in diameter and 0.5 cm in height. The mechanical strength and adhesive forces of the surface of the two gelatin-based hydrogels were measured by changing the experimental probe of a universal testing machine (UTM) (Fig. 6a and S12†). Given that eMNP-3s displayed a low degree of crosslinking, the eMNP-3–gelatin hydrogel was not expected to have a high content of covalent bonding networks such as C–C bonds, C–N bonds, and C–S bonds.^51^ The mechanical strength of the gelatin–eMNP-3s hydrogel was improved by only about 3.1% compared to that of the gelatin alone hydrogel as the control (Fig. 6b). In contrast, the adhesion force of the gelatin–eMNP-3s hydrogel increased by 3.2 fold after its compression for 10 s (Fig. 6c), confirming our hypothesis that the stickiness of the hydrogel containing eMNP-3s was greatly enhanced due to their high contents of catechol-quinone and protonated primary amine groups under the acidic conditions.

## Conclusion

MNPs synthesized *via* the enzymatic method, especially using tyrosinase, can be made from various single monomer substrates such as dopamine and DOPA and monophenolic substrates. The use of enzymes for the synthesis of MNPs allows broad substrate specificity and establishes a green process, but it is not easy to control the one-step synthesis of homogenous MNPs only using a free enzyme. Most melanins produced by enzyme reactions such as tyrosinase, laccase, and peroxidase can easily bind to the surrounding cell extract proteins or chelate metal ions. Consequently, homogenous MNPs of one size cannot be obtained and their morphology is quite heterogeneous, limiting their further industrial applications. Consequently, there are no clear advantages of the enzymatic synthesis method over the chemical method for the preparation of MNPs, such as simple chemical auto-oxidation of dopamine or DOPA under alkaline conditions.

This study showed that homogenous eMNPs could be synthesized using tyrosinase, especially *Bc*Ty, which showed the optimal reaction conditions at acidic pH of pH 3.0–5.0. The enzymatic method was employed for the synthesis of soluble eMNPs with a size of 5–20 nm using various substrates by adjusting the reaction pH below pH 5.0 but above pH 3.0. The different chemical, structural and instrumental analyses revealed that (i) a significant fraction of dihydroxy moieties is maintained and (ii) the amine group in dopamine is protonated due to its low reaction pH below its p*K*_a_ value in eMNP-3s, which are quite similar to the unique properties of Mfp in an acidic environment. Consequently, the crosslinking between the catechol and quinone monomer derivatives oxidized by tyrosinase was inhibited and the polymerization rate was reduced, slowing the growth of the particle size. As the synthetic pH decreased to 3.0, the particle size of eMNPs also decreased, but their solubility was enhanced. Besides, based on the chemical characteristics of the functional groups in the acidic condition and the broad substrate specificity of the tyrosinase reaction, we could synthesize soluble MNPs using various monophenolic substrates. According to our hypothesis, eMNP-3s preserved a high ratio of catechol to quinone moieties, which displayed substantial antioxidant properties and caused the stickiness of the eMNP-3–gelatin hydrogel to increase. Consequently, we demonstrated the synthesis of homogenous eMNPs through a biocompatible one-step synthesis method. We look forward to expanding the applications of eMNPs as antioxidants, surface coatings, imaging reagents, and electronic materials.

## Author contributions

B.-G. K and designed the original idea and supervised the research. H. K. performed the experiments and processed data. B. G. K and H. K prepared the paper. U.-J. L., H. B. S., J. C. L., and H. W. N. conceptualized the idea and discussion the manuscript. W.-S. S. provided guidance for MALDI-TOF analysis. M.-H. K. performed by transmission electron microscopy under the J. W. P. supervised. N. S. H. provided guidance for hydrogel synthesis. B.-S. K performed Universal Testing Machine analysis. All authors read and approved the final paper.

## Conflicts of interest

There are no conflicts to declare.

## Supplementary Material

RA-012-D2RA01276F-s001
