# Supplementary material for: Synthesis of soluble melanin nanoparticles under acidic conditions using Burkholderia cepacia tyrosinase and their characterization
Source: RSC Adv. 2022 Jun 13;12(27):17434–42. doi: 10.1039/d2ra01276f (PMC9189705; doi:10.1039/d2ra01276f)
Supplement: RA-012-D2RA01276F-s001 [file RA-012-D2RA01276F-s001.pdf]

Electronic Supplementary Material (ESI) for RSC Advances.
This journal is © The Royal Society of Chemistry 2022

Supporting Information

Synthesis of soluble melanin nanoparticles under acidic condition using *Burkholderia cepacia* tyrosinase and their characterization

Hyun Kim[1,2], Uk-Jae Lee[1,2], Hanbit Song[1,2], Jeongchan Lee[1,2], Won-Suk Song[1,2], Heewon Noh[1,2], Min-Ho Kang[1,6], Beom-Seok Kim[3], Jungwon Park[1,6], Nathaniel S. Hwang[1], and Byung-Gee Kim[1,2,3,4,5*]

[1]School of Chemical and Biological Engineering, Seoul National University, Seoul, 08826, Republic of Korea

[2]Institute of Molecular Biology and Genetics, Seoul National University, Seoul, 08826, Republic of Korea

[3]Interdisciplinary Program for Biochemical Engineering and Biotechnology, Seoul National University, Seoul, 08826, Republic of Korea

[4]Bio-MAX/N-Bio, Seoul National University, Seoul, 08826, Republic of Korea

[5]Institute for Sustainable Development (ISD), Seoul National University, Seoul, 08826, Republic of Korea

[6]Center for Nanoparticle Research, Institute for Basic Science (IBS), Seoul 08826, Republic of Korea

Table S1. Primer list

Primer	Sequence (5’ → 3’)
***Bc*Ty _F**	GGAGATATACCATGGCAAATAACGCATCTGGAGTCAG
***Bc*Ty _R**	CGGCGGCAAGCTTTCGGACCTCGAGCCGGA
***Bc*Ty-pET28a-CPEC_F**	GTCCGAAAGCTTGCGGCCG
***Bc*Ty-pET28a-CPEC_R**	AGATGCGTTATTTGCCATGGTATATCTCC

	Optimum pH	Substrate	V_{max} (μmol·min^{-1}·mg^{-1})	K_m (mM)	k_{cat} (s^{-1})	k_{cat}/K_m (mM·s^{-1})
Agaricus bisporus Ty (Fenoll, Rodríguez-López et al. 2001)	6	L-Tyrosine	10.8	0.25 ± 0.03	7.9 ± 0.12	31.60
		L-DOPA	145.9	0.28 ± 0.01	107.3 ± 1.45	383.21
Bacillus megaterium Ty (Deri, Kanteev et al. 2016)	8	L-Tyrosine	3.62 ± 0.06	0.082 ± 0.006	2.1	25.60
		L- DOPA	30.3 ± 0.6	0.24 ± 0.02	17.8	74.20
Streptomyces avermitilis Ty (Lee, Lee et al. 2015)	7	L-Tyrosine	1.05 ± 0.037	0.589 ± 0.056	0.021	0.04
		L- DOPA	9.67 ± 1.85	2.79 ± 0.79	0.19	0.07
Burkholderia thailandensis Ty (Son, Lee, Lee et al. 2018)	5	L-Tyrosine	404.24 ± 18.88	0.59 ± 0.055	397.5	668.63
		L- DOPA	495.42 ± 44.66	0.83 ± 0.13	487.17	586.74
Burkholderia cepacia Ty (This study)	4	L-Tyrosine	335.58 ± 31.49	0.22 ± 0.04	333.9 ± 31.33	1504.94
		L- DOPA	1812.12 ± 299.35	2.16 ± 0.41	1803.06 ± 297.9	835.42

Table S2. Kinetic parameters of tyrosinases (Ty).

Note. DOPA: dihydroxyphenylalanine

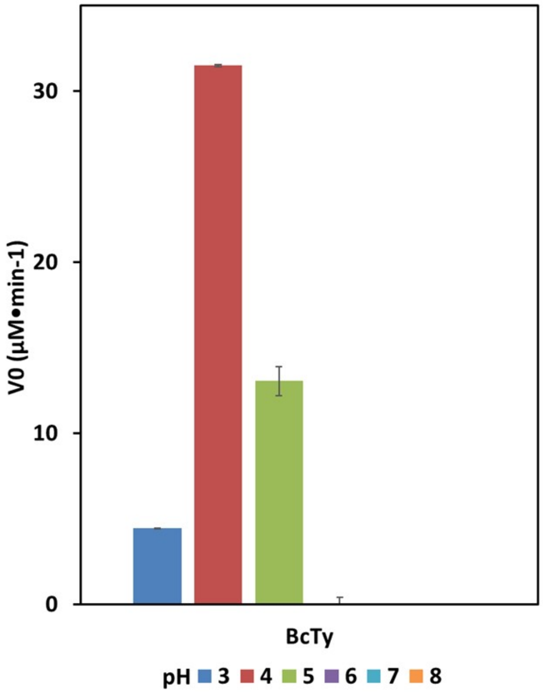

Figure S1. Optimal pH of *Bc*Ty activity. Assay was performed by measure a conversion of l-tyrosine by *Bc*Ty under each 50 mM buffer (pH 3.0, 4.0, 5.0: citric buffer, pH 6.0, 7.0: phosphate buffer, pH 8.0: Tris buffer). For the assay, 100 µl of 400nM *Bc*Ty solution with 10 µM of $CuSO_4$ was mixed with 100 µl of 2 mM L-tyrosine solution. The total reaction volume was 200 µl at 37°C. For initial velocity measurements, UV absorption at 475 nm (ε = 3600 $M^{-1}cm^{-1}$) was recorded every 1 min by a UV spectrometer for a total of 30 min. The initial velocity of *Bc*Ty (V_0) was calculated by plotting the initial 5 points of the response, based on the triplet set of the experiment.

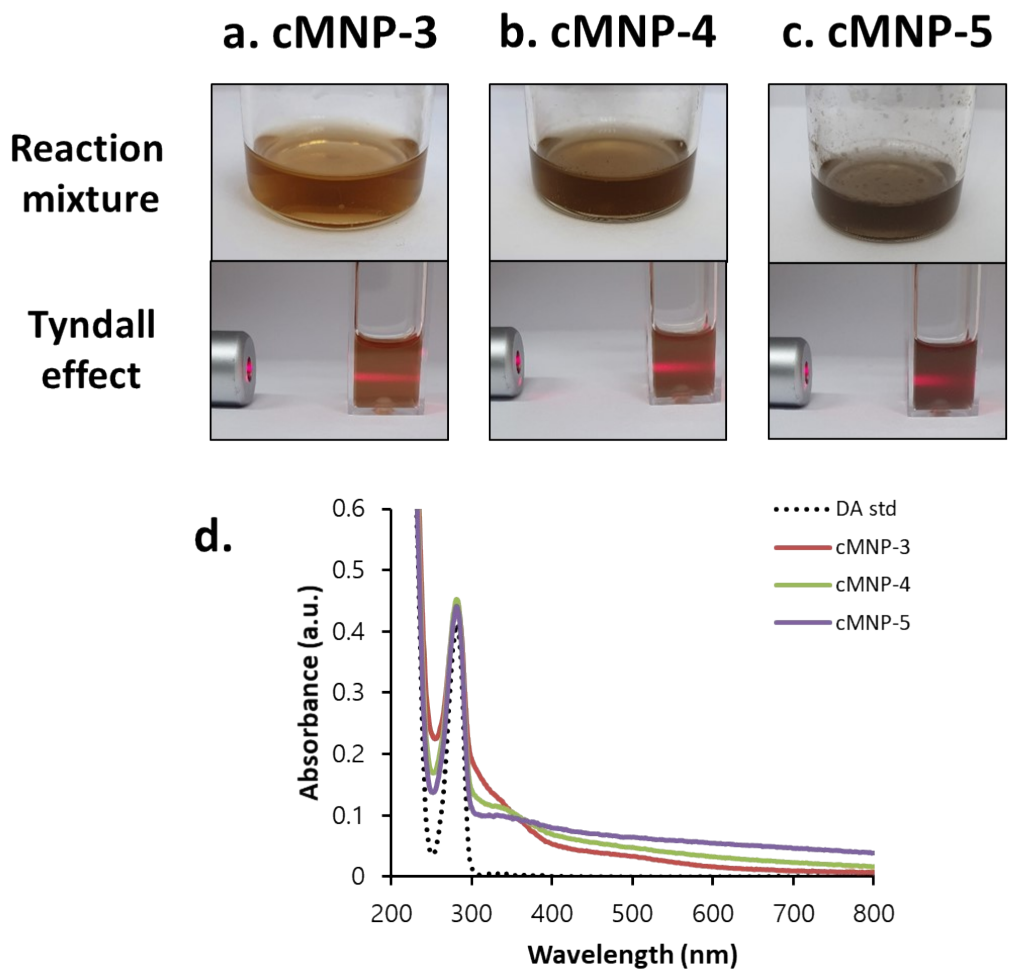

Figure. S2 Digital pictures of melanin reaction solutions and Tyndall effect by irradiating 660 nm red laser synthesized from 1 mg/ml of dopamine using various synthesis methods and their scanning electron microscopy images; (a) pH 3.0 - APS, (b) pH 4.0 - APS, (c) pH 5.0 – APS. (d) UV spectrum profile of cMNP-3, 4, and 5. The black dot line represents the dopamine 3 mg/ml standard dissolved in DW. The samples for UV sepctrum were diluted with water from 3 mg/ml to 0.05 mg/ml.

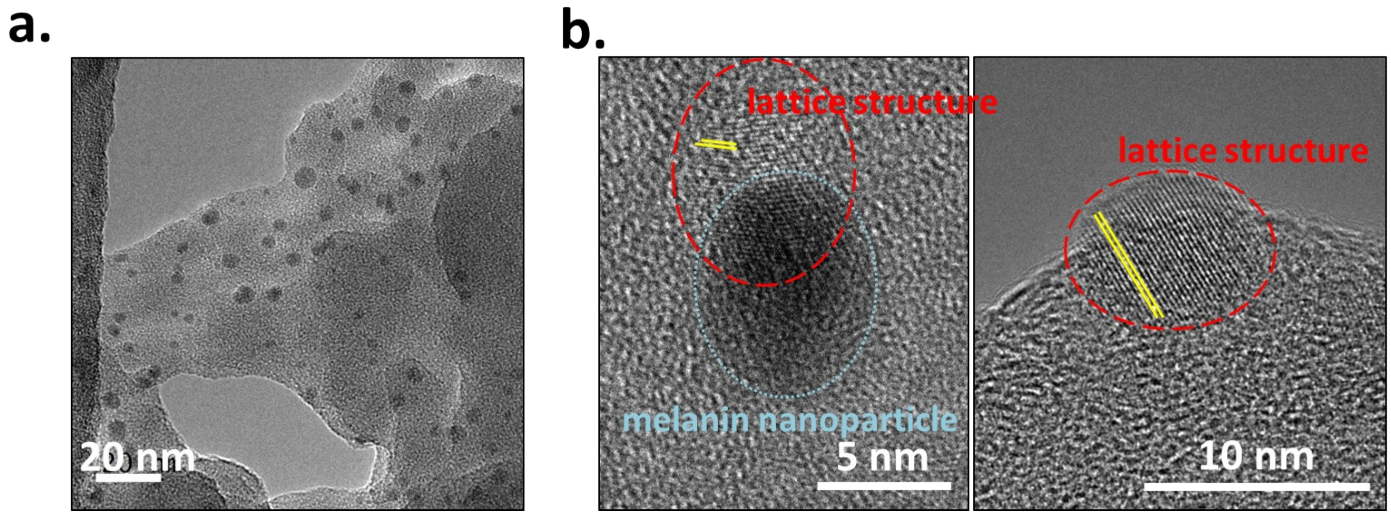

Figure. S3 TEM image of (a) eMNP-3 and (b) Nano-onion like stacking structure of eMNP-3. The blue dot line represents melanin nanoparticles, and the red dot line shows the corresponding onion-like stacking structure. The length of each gap between the lattice is approximately 0.3 nm (Yellow line shows the gap between lattice.).

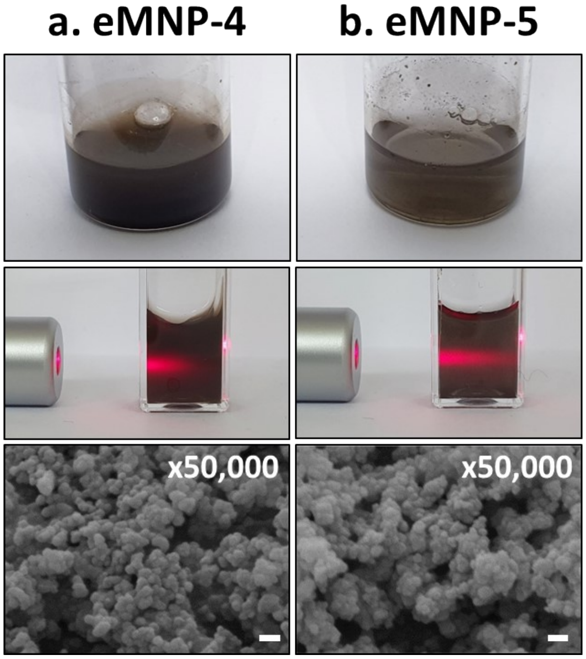

Figure. S4 Digital picture of melanin reaction solution and Tyndall effect by irradiating 660 nm red raser synthesized from 3 mg/ml of dopamine at acidic conditions. And their scanning electron microscopy image; (a) pH 4 - *Bc*Ty, (b) pH 5 – *Bc*Ty. Scale bar: 200 nm. Average hydrodynamic diameters of (a) eMNP-4 and (b) eMNP-5 measured by DLS. The sample for the tyndall effect and DLS was diluted with water from 3 mg/ml to 0.05 mg/ml.

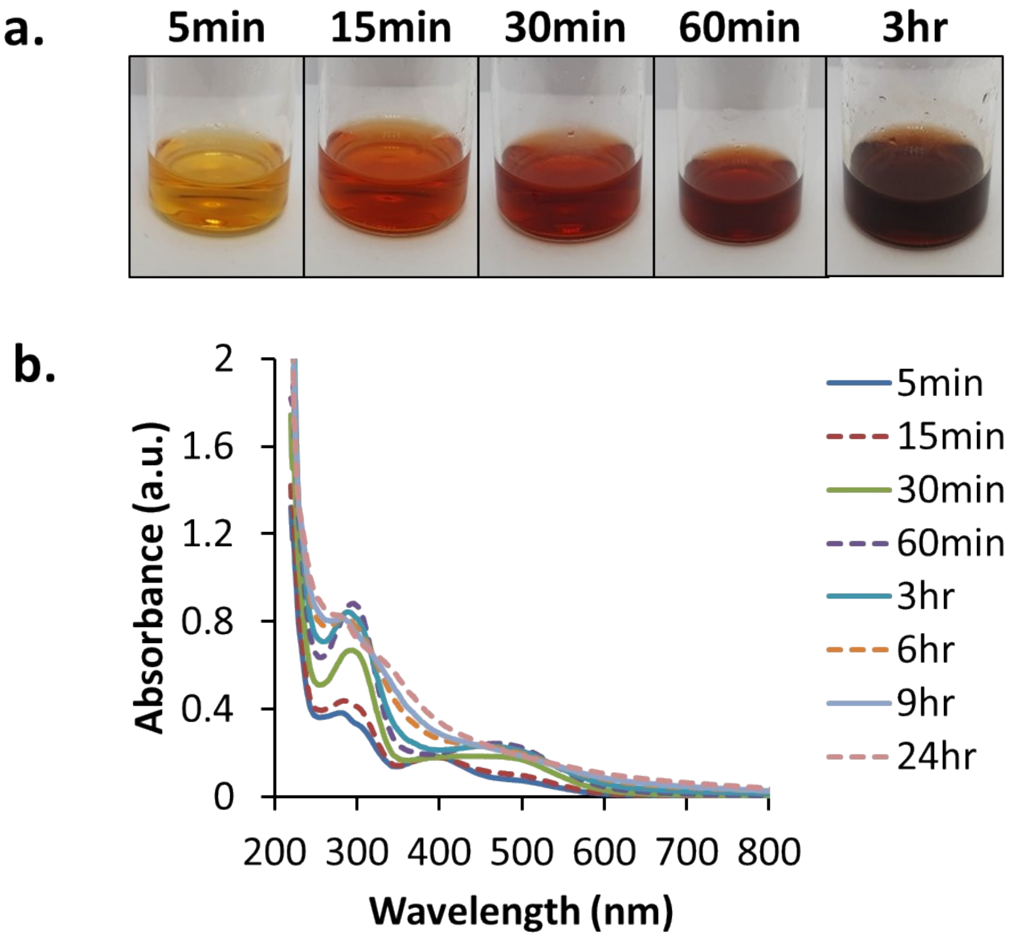

Figure. S5 (a) Time point image of eMNP-3 reaction solution. (b) UV spectrum profile (220 – 1000 nm) change of the eMNP-3 reaction mixture over time and representative digital picture corresponding to each time points

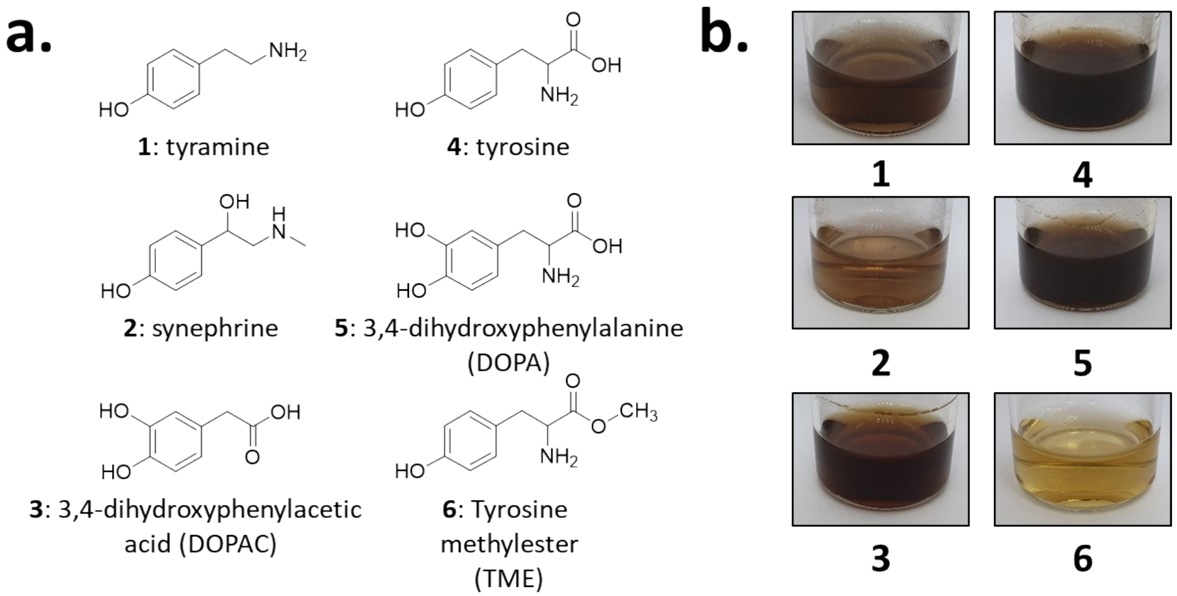

Figure. S6 (a) Chemical structure of eMNP substrates based on the phenolic compound and (b) representative digital image of produced soluble eMNPs.

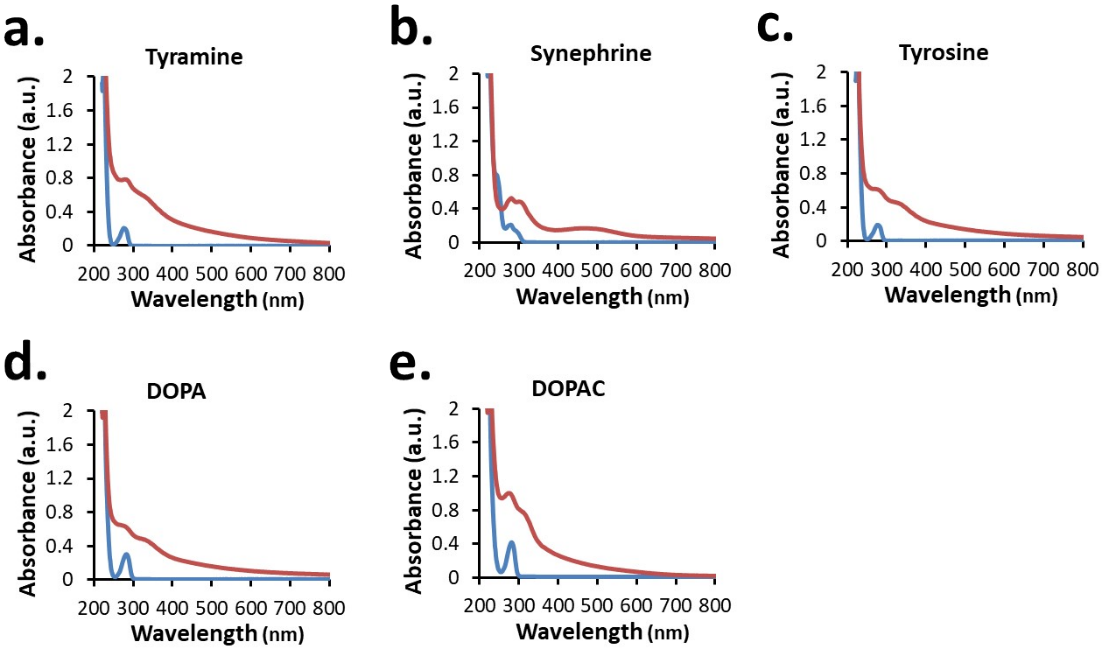

Figure. S7 UV spectrum profile between eMNP synthesized according to substrate variation - (a) tyramine, (b) synephrine, (c) tyrosine, (d) DOPA, (e) DOPAC - and substrate standard. Blue line indicates substrate standards; red line indicates eMNPs. All sample were diluted with water from 3 mg/ml to 0.05 mg/ml before UV spectroscopy.

Chemical structure	m/z
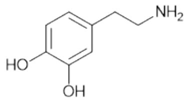	**154.09**
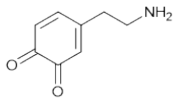	**152.07**
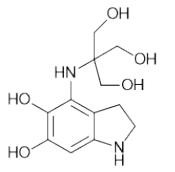	**273.20**

Chemical structure	m/z
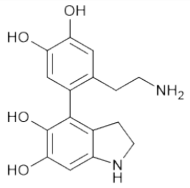	**305.34**
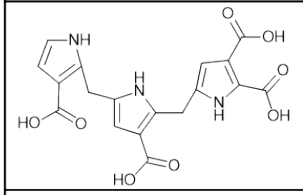	**152.07**
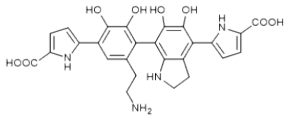	**273.20**

Figure. S8 The predicted chemical structure of dopamine oligomeric derivatives using positive mode of MALDI-TOF

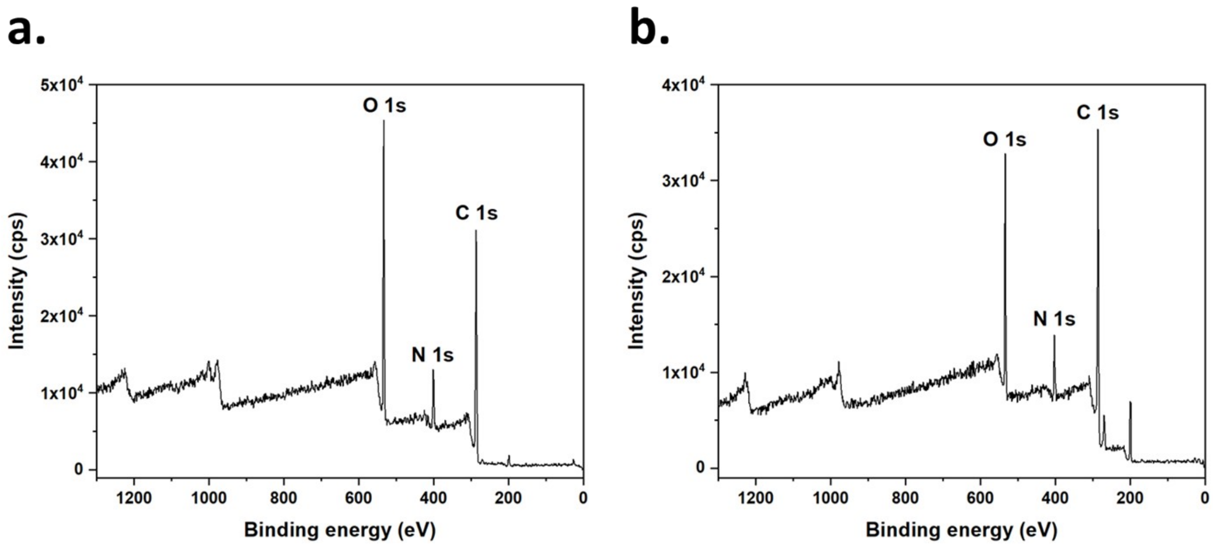

Figure. S9 XPS survey spectrum of (a) eMNP-3 and (b) cMNP

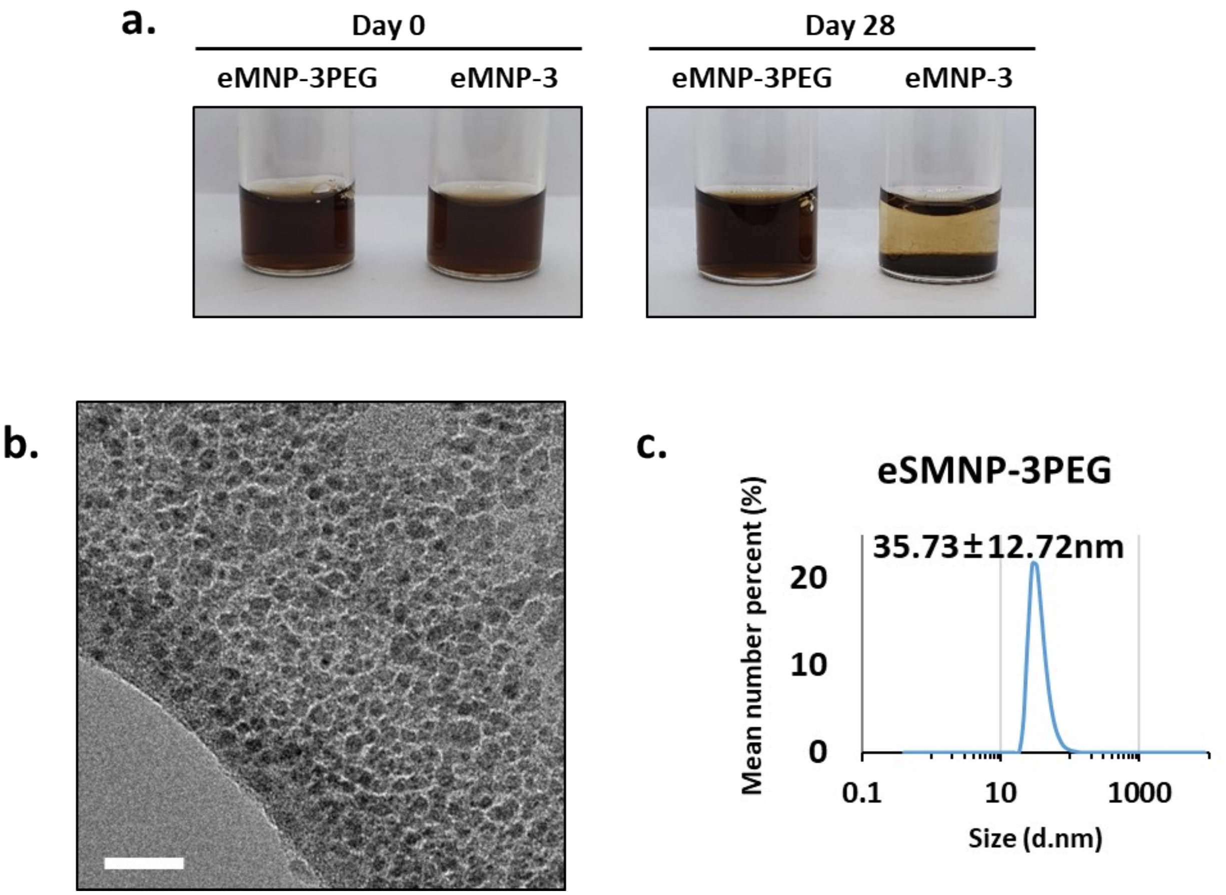

Figure. S10 Surface modification of soluble eMNP. (a) PEG-modified eMNP (eMNP-3PEG) stably dispersed in phosphate-buffered saline (PBS) over 4 weeks. (b) TEM image and (c) hydrodynamic diameter of eMNP-3PEG. Scale bar: 20 nm. The sample was diluted with water from dopamine concnetration of 3 mg/ml to 0.05 mg/ml.

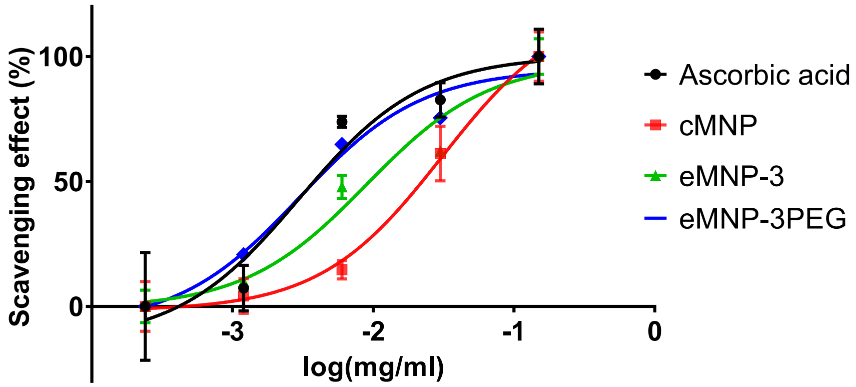

Figure. S11 Comparison of radical scavenging activity (%) between MNPs and ascorbic acid. The antioxidant activity of eMNP was measured in PBS buffer (pH 7.4) to mimic the physiological condition with EC_{50} for the antioxidant activity required to reduce the concentration of 0.1 mM DPPH, a free radical generator, by 50%.

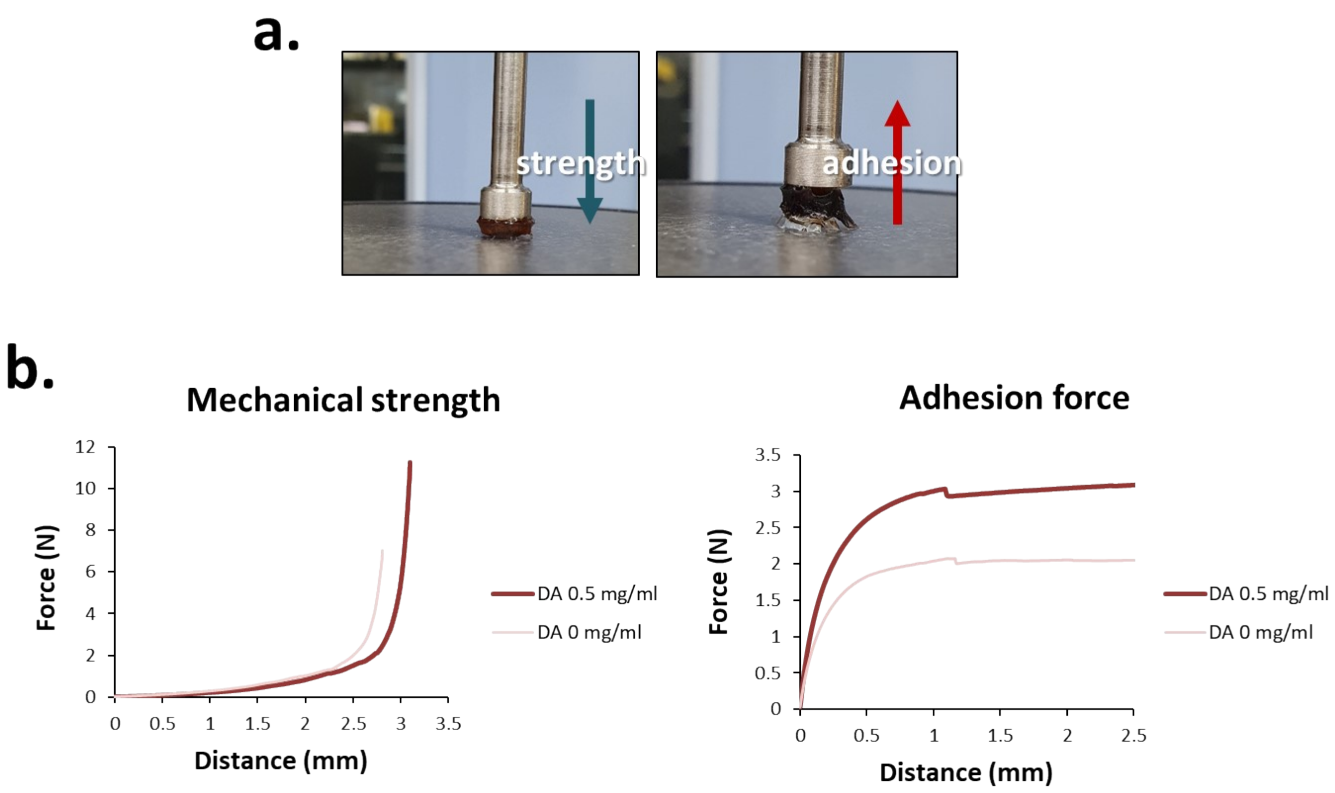

Figure. S12 (a) Schematic image of the mechanical strength and adhesion force of melanin hydrogel. (b) Representative stress-strain curve
